# 3D morphological analysis of the mouse cerebral vasculature: Comparison of *in vivo* and *ex vivo* methods

**DOI:** 10.1371/journal.pone.0186676

**Published:** 2017-10-20

**Authors:** Joe Steinman, Margaret M. Koletar, Bojana Stefanovic, John G. Sled

**Affiliations:** 1 Mouse Imaging Centre, The Hospital for Sick Children, Toronto, Ontario, Canada; 2 Department of Medical Biophysics, University of Toronto, Toronto, Ontario, Canada; 3 Sunnybrook Research Institute, Toronto, Ontario, Canada; University of Arizona, UNITED STATES

## Abstract

*Ex vivo* 2-photon fluorescence microscopy (2PFM) with optical clearing enables vascular imaging deep into tissue. However, optical clearing may also produce spherical aberrations if the objective lens is not index-matched to the clearing material, while the perfusion, clearing, and fixation procedure may alter vascular morphology. We compared *in vivo* and *ex vivo* 2PFM in mice, focusing on apparent differences in microvascular signal and morphology. Following *in vivo* imaging, the mice (four total) were perfused with a fluorescent gel and their brains fructose-cleared. The brain regions imaged *in vivo* were imaged *ex vivo*. Vessels were segmented in both images using an automated tracing algorithm that accounts for the spatially varying PSF in the *ex vivo* images. This spatial variance is induced by spherical aberrations caused by imaging fructose-cleared tissue with a water-immersion objective. Alignment of the *ex vivo* image to the *in vivo* image through a non-linear warping algorithm enabled comparison of apparent vessel diameter, as well as differences in signal. Shrinkage varied as a function of diameter, with capillaries rendered smaller *ex vivo* by 13%, while penetrating vessels shrunk by 34%. The pial vasculature attenuated *in vivo* microvascular signal by 40% 300 μm below the tissue surface, but this effect was absent *ex vivo*. On the whole, *ex vivo* imaging was found to be valuable for studying deep cortical vasculature.

## Introduction

The brain is highly sensitive to reductions in blood flow. Occlusions of arterioles cause microinfarcts, which may coalesce across the cortex if multiple arterioles are occluded [1]. Hypoperfusion in Alzheimer’s disease is associated with white-matter lesion formation and microinfarcts [2]. Flow reductions co-localize with white matter vascular disease and correlate with cognitive function [3, 4]. Cerebrovascular architecture is one of the major determinants of cerebral blood flow (CBF), since the diameters and lengths of individual vessels, and their connections in 3D space, dictate vascular network resistance [5, 6]. Architectural changes, such as stiffening of cerebral arteries or increased vessel tortuosity, are seen in cognitive impairment, enlarged ventricles, and dementia [7, 8].

A variety of imaging techniques exist for visualizing the cerebral vasculature; however, few possess the resolution or tissue imaging depth to visualize arterioles, venules, and capillaries deep into cortex. Micro-computed tomography can image an entire mouse brain in 3D, but the resolution of bench top systems (20 μm) precludes capillary visualization [9]. In contrast, confocal microscopy possesses resolution on the order of 1 μm, but the depth of penetration into tissue is only 100–200 μm due to the high degree of scattering at the wavelengths utilized [10]. Two-photon fluorescence microscopy (2PFM), on the other hand, provides cellular resolution and greater depth penetration. *In vivo* 2PFM provides capillary-resolution images up to 800 μm below the cortical surface, a several-fold improvement over confocal microscopy [11]. It is valuable for imaging microvascular architecture since it possesses sufficient resolution for visualizing capillaries, and allows imaging well below the brain surface. This paper contrasts *in vivo* 2PFM with an alternative 2-photon fluorescence imaging method based on *ex vivo* cleared tissue specimens.

Even though *in vivo* 2PFM provides improvement in resolution and/or imaging depth in comparison with other methods, it is unable to visualize the vasculature through the entire mouse cortex, as it exceeds 1.5 mm thickness in some cases [12]. To enhance depth penetration, *ex vivo* tissue clearing techniques may be combined with 2PFM. Optical clearing has been shown to enhance imaging of brain [13] and spinal cord tissue [14]. High refractive index materials similar to tissue membranes render tissue the most transparent, and provide the deepest imaging. For example, SeeDB, a fructose-based clearing agent, enables an imaging depth up to 8 mm with 2PFM [15].

While optical clearing improves imaging depth, tissue dimensions may be changed by the clearing materials. This in turn may distort morphological measurements. If the refractive index of the clearing agent (and by extension the cleared tissue) differs from that of water (the typical immersion medium of the microscope objective), spherical aberrations will occur. These aberrations worsen with depth, and are the most severe for the higher refractive index materials which result in optimal tissue clarity. To overcome these aberrations, oil-immersion objectives may be used, but these typically have a working distance less than 300 μm, defeating the purpose of optical clearing. Smaller vessels, whose dimensions are on the order of those of the Point Spread Function (PSF) of the imaging system, are proportionately more distorted relative to their diameters. This may result in an overestimation of their diameters, and reduce the ability of computational algorithms to detect and segment these vessels.

The goal of this work is to contrast *ex vivo* and *in vivo* imaging, while highlighting the advantages and disadvantages of each. To achieve this goal, an *ex vivo* 2PFM methodology for mouse cortical vascular imaging is developed and presented. The methodology consists of the following components: (A) perfusing the vasculature with a fluorescent gel that solidifies inside the vasculature, rendering the vessels visible under a fluorescent microscope; (B) clearing the tissue via immersion in a high-concentration fructose solution; and (C) accurate calculation of vessel diameters via a novel segmentation algorithm that accounts for the spherically aberrated and spatially varying *ex vivo* PSF. This *ex vivo* technique is then used to contrast vascular networks imaged *iv vivo* vs *ex vivo*. Mice are imaged with *in vivo* 2PFM; these imaged regions are then identified following tissue processing and imaged *ex vivo*. Identical vessels in both images are identified with advanced registration algorithms, enabling the comparison of the properties (signal, morphology) of corresponding vessels *in vivo*/*ex vivo*. This study assesses *in vivo/ex vivo* imaging beyond well-characterized metrics such as imaging depth, which is possible through the advanced registration and image analysis techniques utilized.

## Materials and methods

### *In vivo* imaging

*In vivo* procedures were approved by the Animal Care Committee of the Sunnybrook Health Sciences Centre, under Animal Use Protocol 15–563. Four mice total (C57BL/6) were imaged on separate days, with both *in vivo* and *ex vivo* 2PFM. Mice were anaesthetized via isoflurane [5% induction, 1.5–1.75% maintenance in oxygen enriched medical air (30% oxygen, balanced nitrogen)] as described in Dorr et al. [8]. The oxygen supplement was to compensate for the respiratory depression effect from general anesthesia. A stereotaxic frame with a bite bar and ear bars restrained the mice. Core body temperature was maintained at 37 ±3 oC using a rectal probe and a feedback-controlled heating pad (TC-1000, CWE Inc.). Other physiological parameters were monitored by pulse oximetry (MouseOx Plus) for a target heart rate of approximately 500 beats per minute, respiration rate 60–80 breaths per minute, and oxygen saturation 98 ± 1%. Since mice were not intubated (or maintained by mechanical ventilation), end expiratory carbon dioxide was not measured. Stereotaxic surgery was performed, the primary somatosensory cortex was demarcated, and a small (< 5 mm diameter) cranial window drilled in this area. This window was sealed with a 5 mm diameter coverslip (World Precision Instruments) glued to the skull.

2PFM was performed using a twin FV 1000 Multi Photon Excitation Microscope (Olympus Corp., Tokyo, Japan) with a 2 mm working distance water immersion microscope objective (25 x, 1.05 NA). An intraperitoneal (IP) injection of the fluorescent contrast agent SR101 (8 μL/g of body weight) dissolved in 0.1 M phosphate buffered saline (PBS) was administered [16]. Images were acquired at 0.994 μm lateral resolution and 1.5 μm axial step size. The excitation wavelength was 900 nm. The objective used for this high-resolution 3D imaging was replaced with a 5 x 0.1 NA objective to obtain a map of the pial vasculature on the cortical surface. This image possessed a 2.5 mm field of view, in comparison with the 0.5 mm field of view for the for the 1.05 NA objective, and was obtained at 5 μm nominal lateral resolution with a 50 μm step size. This image was later used to identify the same imaging region *ex vivo* as *in vivo*.

Since the SR101 dye has a small molecular size, there is a possibility that it penetrates the glycocalyx, unlike the FITC albumin dye. If this were the case, *in vivo* diameters could be overestimated relative to *ex vivo*. To assess whether or not this had occurred, an additional mouse was imaged *in vivo* on the same 2PFM system. This mouse was simultaneously administerd an IP SR101 injection as previously described, as well as a tail vein injection of a 2 MDa FITC dextran which is too large to pass into the glycocalyx. The signal from the red and green dyes were separated using 495–540 and 575–630 bandpass filters. Both dyes were excited at 900 nm, and imaging was performed with the 25 x objective, at 0.994 μm lateral resolution and 1.5 μm axial step size.

### *Ex vivo* animal preparation and imaging

Following *in vivo* imaging, mice were removed from the stereotaxic frame, and anaesthetized a 2^nd^ time with an IP injection of 100 μg ketamine per gram of body weight (Pfizer, Kirkland, QC, Canada) and 20 μg xylazine per gram of body weight (Bayer Inc., Toronto, ON, Canada). They were transferred to the fumehood, where an incision was made in the chest to allow for opening of the chest cavity, and a 24-gauge IV catheter (Becton Dickinson Infusion Therapy System Inc., UT, USA) was inserted into the left ventricle. A slit made in the right atrium permitted outflow of blood. Mice were perfused at a constant volume flow rate of 5 mL/min with 30 mL of heparinized (5U/mL) 0.1M PBS (Wisent Inc., St-Bruno, QC, Canada), 30 mL of 4% paraformaldehyde (PFA) (Electron Microscopy Sciences, Hatfield, PA, USA), and 20 mL of a 2% (w/v) gelatin solution (Sigma, St. Louis, MO, USA) combined with 1% (w/v) FITC-conjugated albumin (FITC-albumin; Sigma) [17]. Prior to perfusion, the gelatin solution was filtered through 0.8 μm pore size syringe filter.

**Fig 1:**
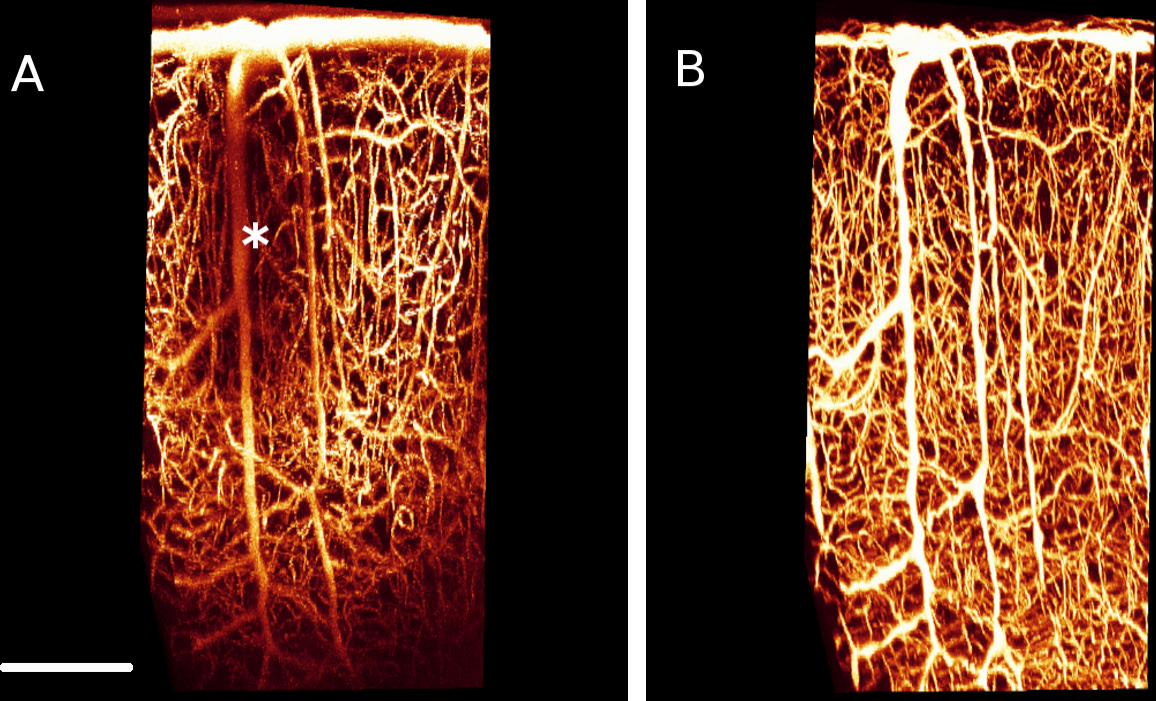
Maximum Intensity Projections (MIPs) of corresponding *in vivo-ex vivo* datasets from a single mouse. (A) *In vivo* MIP. (B) *Ex vivo* MIP. Both A and B display the vasculature with cortical depth. Unlike in the *ex vivo* image, where a relatively constant signal is maintained through the cortical depth, the signal *in vivo* is relatively weak at about 400–500 μm below the cortical surface. In addition, vessels *in vivo* beneath the pial vasculature (large diameter vessels at the cortical surface) are detected at a weaker signal compared to those that are not underneath these vessels (see the dark patch demarcated by the * in panel A). Scale bar = 0.2 mm.

Following perfusion, mice were decapitated and the skin remove from the skull. The brains (while inside the skull) were immersion-fixed for 24 hours in 20 mL of 4% PFA. The coverslip was removed, and the tissue was cleared in graded solutions at 12 hours each of 20%, 40%, 60%, 80%, and then 100% SeeDB (80.2% wt/wt fructose in distilled water with 0.5% α-thioglycerol) [15] while inside the skull. Brains were removed from the skull following clearing, and were glued to the inside of a glass container. This container was filled with SeeDB, and sealed with a No. 1 glass coverslip. A drop of water dripped onto the coverslip surface enabled the 1.05 NA water-immersion objective lens to be dipped into the water-drop. Images were obtained at 800 nm excitation, and encompassed the identical region that was imaged *in vivo*. Lateral pixel size was the same (0.994 μm) as that *in vivo*, while the apparent axial step size was 1.34 μm. However, an apparent step size of 1.34 μm corresponds to an actual step size of 1.5 μm (as per the *in vivo* image), since the refractive index mismatch between the clearing agent and water causes the apparent axial step to be scaled by a factor of 1.12 (scaling factor = n_agent_/n_water_ = 1.49/1.33 = 1.12). Multiple images of overlapping volumes were acquired to maximize the likelihood that vessels imaged *in vivo* were captured *ex vivo*. Image stitching was performed using the algorithm described in Emmenlauer et al. [18] to produce a single large field-of-view image. Fig 1A and 1B below is an example of a corresponding *in vivo/ex vivo* image pair.

### Image analysis

In comparing vascular architecture *in vivo* and *ex vivo*, the vessels in both images were automatically segmented with an in-house developed vessel tracking software [19] (see Vessel tracking). No manual correction of the segmented data was required, and all post-processing was performed automatically. Registration and identification of common vessels in both images enabled a 1-to-1 correspondence to be made between *in vivo*/*ex vivo* vessels for the comparison analysis (elaborated upon in below).

#### Vessel tracking

Vessel tracking (the algorithm used here for segmenting the vasculature) differs from traditional binarization segmentation techniques since it traces the centerlines of vessels that are approximated as discrete medial atoms [20], as opposed to binarizing and iteratively thinning the binarized image. At discrete points along the vessel (vertices), the centerline is sampled through optimization of the radius and position in 3D space of an image operator. This operator is defined by 8 spokes extending from the vessel centre (ie. medial atom) to its boundary. This procedure enables identification of the centerline location, local tangent vector to the vessel, and the vessel radius. The metric that is optimized is the sum of the intensity gradients measured for each spoke. A low gradient calculated at the tip of a particular spoke indicates a potential branch point.

Each xy-plane in the *in vivo/ex vivo* images was convolved with a 2D Gaussian blurring kernel with a full-width-half-maximum (FWHM) of 1.5 μm. The image was resampled to an isotropic voxel size of 1.5 x 1.5 x 1.5 μm^3^, after which a non-local means denoising filter was applied [21].

On the order of 400 000 seeds (digital markers)/mm^3^ were automatically placed inside the vessels of an image to initiate tracking. Despite thousands of seeds being created, the tracking time was increased by a negligible amount since seeds in previously tracked vessels were ignored by the algorithm. It is necessary to include multiple seeds throughout an image (instead of initiating tracking with a single seed inside a single vessel) as this ensures all vessels are segmented, including those partially contained within the field of view that may appear disconnected from the rest of the network. Seed locations were determined separately for each image by manually selecting a signal threshold to separate vessels in the foreground (which possess a large signal) from the weak tissue background. Voxels that were a local signal intensity maxima with respect to their six nearest neighbours were chosen as seed locations. Because the centerline of a vessel often corresponds to a local maximum in signal intensity, this algorithm ensures that a marker is placed close to the center of the vessel.

**Fig 2:**
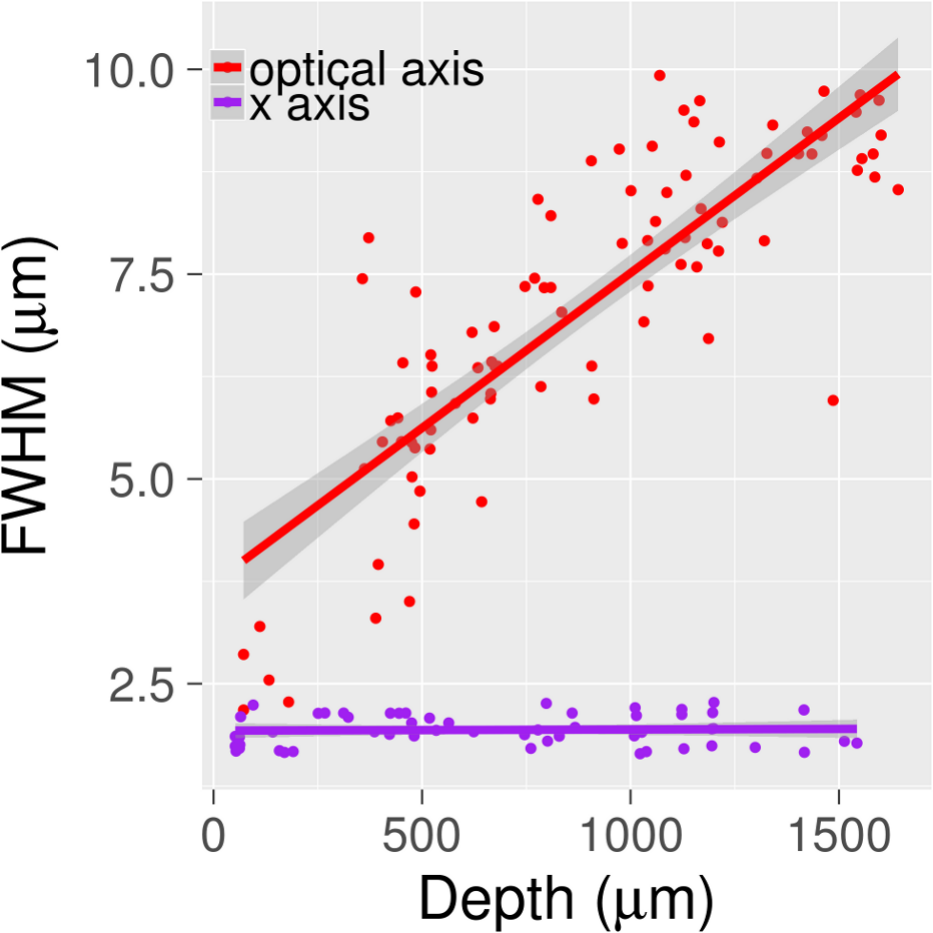
FWHM of signal along optical axis and x-axis versus depth for beads embedded in agar. The beads were 0.5 μm diameter yellow-green fluorescent beads (excitation peak 505 nm; emission peak 515 nm) and were embedded in fructose-cleared 1% low melting point agar. Imaging was performed using 2PFM at an excitation wavelength of 800 nm. The FWHM was calculated by fitting a Gaussian to the signal profile along either the optical or x-axis for these beads. Prior to fitting the Gaussian, the image of the beads was blurred by a Gaussian with FWHM 1.5 μm, as per the vascular images on which vessel tracking was performed. Since the slope of the x-axis was not statistically different from 0 (p = 0.8136), only the PSF along the optical axis was assumed to change with depth when performing vessel tracking. The ribbons surrounding the straight lines represent the 95% confidence interval.

The anisotropic PSF of the 2PFM data was approximated as a 3D Gaussian distribution, with different FWHM values within the xy-plane and through the z-axis. In tracing vessels, the marginal distribution of the PSF in the plane perpendicular to the vessel axis was computed. This was convolved with the proposed circular cross-section of the vessel, approximated as a 2D Gaussian distribution. These convolved distributions (marginal distribution of the PSF and circular cross section) defined an ellipse in the given plane. The procedure outlined above for the circular image operator described in Rennie et al. [19], involving the computation of the gradient at the 8-spoke tips, was repeated to calculate the vessel radius, tangent vector, and centerline. As shown in Fig 2, the refractive index mismatch between fructose and water causes the axial width of the PSF to increase linearly with tissue depth *ex vivo*. The PSF dimensions were assumed to be constant when tracking the *in vivo* data. As per the straight line fit in Fig 2, the axial PSF was specified in vessel tracking to increase in a linear manner from 3.4 μm at the surface of the tissue to 7.5 μm at 1 mm depth. The axial FWHM for the *in vivo* image was maintained constant through the cortical depth at 3.4 μm. The FWHM along the x- and y-axes perpendicular to the optical axis was assumed to be unchanged with depth for both image types (*in vivo* and *ex vivo*).

During tracking, fluctuating voxel intensities caused by noise on the vessel surface may be transformed into short free-ends ('hairs') emanating from the centerlines of actual vessels. A hair was defined as a free-end vessel segment with length not exceeding the radius of the vessel to which it is connected by more than an empirically selected amount of 8 μm. All such vessels were removed.

#### Image registration

Identification of common landmarks corresponding to pairs of identical vessels, such as vascular branch points, enabled *in vivo-ex vivo* images to be registered to one another via a thin plate splines algorithm (TPS) [22]. This same transformation was applied to the *ex vivo* trees generated via tracking the *ex vivo* data. This aligned the *ex vivo* trees with the *in vivo* data in *in vivo* image space, and the vessel diameters of these trees were recalculated in *in vivo* space as described in the section Vessel tracking. The diameter calculation was performed using the same algorithm described above. Since the calculations were in *in vivo* space, the PSF width was assumed to be constant with depth. This procedure enabled each vertex in the original *ex vivo* tracing to be correlated with the same vertex in *in vivo* space, thereby allowing a direct comparison between properties of the same vessel *in vivo*-*ex vivo*. To compare the diameters/signal of corresponding vessels it is also possible to apply the inverse of the transformation just described to the *in vivo* tree, and recalculate the diameters in *ex vivo* space. The option was chosen to recalculate diameters in *in vivo* space (ie. apply the transformation to the *ex vivo* tree) due to the strong signal maintained through the cortical depth in the *ex viv*o images, and the lack of shadowing artifacts *ex vivo* (discussed in further detail in the Results and Discussion sections).

To evaluate the success of the gelatin perfusion on a vessel-by-vessel basis, the above transformation applied to the *ex vivo* tree was inverted and applied to the original tracing of the *in vivo* tree in *in vivo* space. This resulted in the *in vivo* centrelines being aligned to the *ex vivo* image. Manual selection of a background threshold for the *ex vivo* image was performed, and the contrast-to-noise ratio (CNR) at each vertex was calculated by subtracting the mean background signal from the vertex signal, and dividing this result by the standard deviation of all background voxel signals. A vessel was categorized as unperfused if more than 50% of the CNR values for all vertices in the vessel approached zero.

#### Extracting quantitative parameters for comparisons between *in vivo/ex vivo* networks

The capillary bed possesses a net-like architecture, while the arteriolar and venular structure is tree-like [23]. Analyses of vascular architecture often separate larger from smaller diameter vessels [23]. A capillary for this study was defined as a vessel segment with a diameter less than 8 μm that was not defined as a penetrating vessel (see below). A threshold of 8 μm was selected based on the inflection point in the histogram of diameter distributions as proposed in Risser et al. [24]. A segment was defined as the part of a network between either of two bifurcations, or between a bifurcation and non-connected segment end, or between two non-connected segment ends.

Penetrating vessels were extracted from the vascular tree by visually examining the tree and placing a marker inside the part of a penetrating vessel determined to be closest to the cortical surface. This vessel was traced downwards starting from the marker. At each bifurcation, the tracing algorithm followed the vessel segment making the smallest angle with the normal to the cortical surface. Tracing of a penetrating vessel ceased upon the marker reaching the end of a vessel segment not connected to any vessel below it. Penetrating arterioles were identified as those vessels having relatively few branches across the cortical depth, a relatively constant diameter, and are surrounded by a capillary free-space. Penetrating venules, in contrast, were identified as those with more branches, a smaller capillary-free space, and an increasing diameter moving towards the cortical surface [25, 26].

The radius at each vertex of the centerline network was computed. To calculate the radius of a vessel segment, the radii of all vertices comprising the segment were averaged. The vessel signal was defined as the mean of all voxel signals within that segment.

## Results

**Fig 3:**
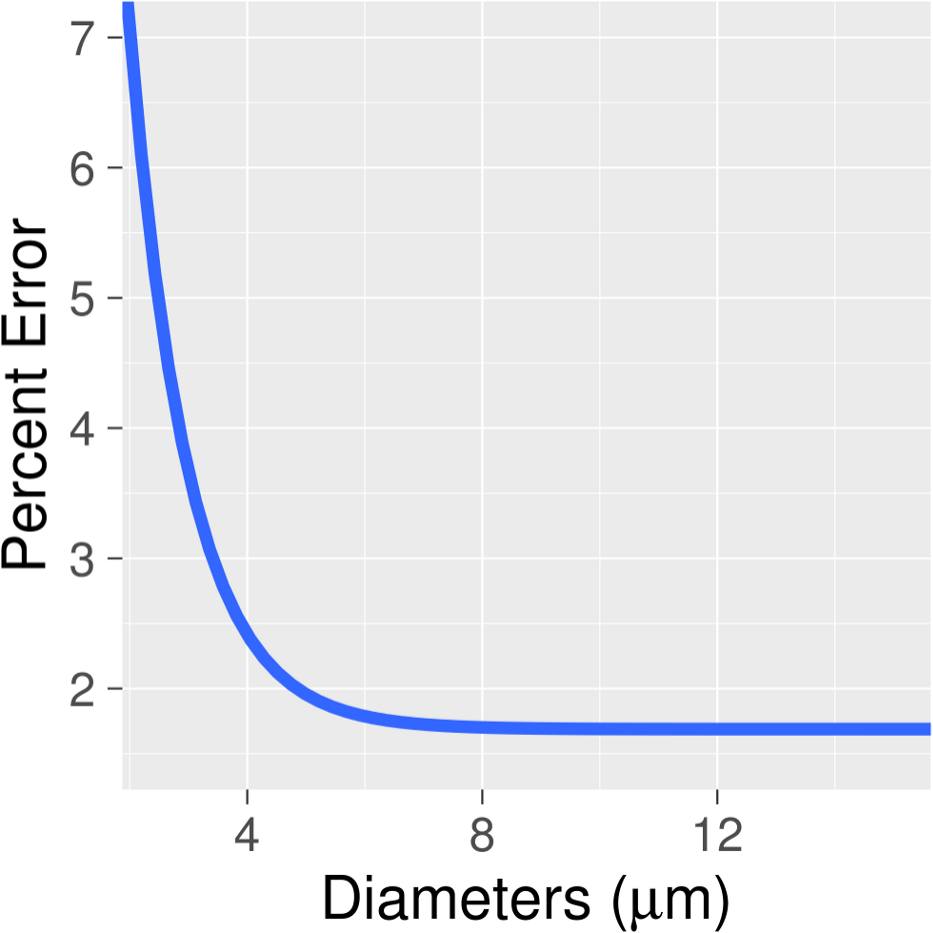
Percent error in diameter estimation as a function of vessel diameter. For this figure, an *ex vivo* image was tracked assuming a spatially varying PSF, using values from the straight line fits in Fig 2. Diameters were then recalculated for each tracked vertex assuming an unchanging PSF-width with image depth. The percent difference on this plot is the percent difference between the diameter calculated while accounting for a changing PSF, and that calculated without accounting for this spatial variance. The x-axis is the diameter calculated assuming a changing PSF (ie. the diameter initially calculated). The line displayed is an exponential fit to the data. For the small vessel diameters, where the size of the PSF is close to that of the vessel, the percent difference is appreciable. For vessels with diameters above 5 μm, this effect is much smaller (<2%).

Accounting for the spatially varying PSF in the *ex vivo* data had the greatest effect for smaller vessels less than 5 μm in diameter. This resulted in an error in diameter calculation of up to slightly greater than 7% for these vessels (see Fig 3). The effect of incorporating the spatial variance of the PSF was very small (error reduction less than 2%) for the vessels with diameters larger than the PSF z-extent (about 5 μm) (see Fig 3).

**Fig 4:**
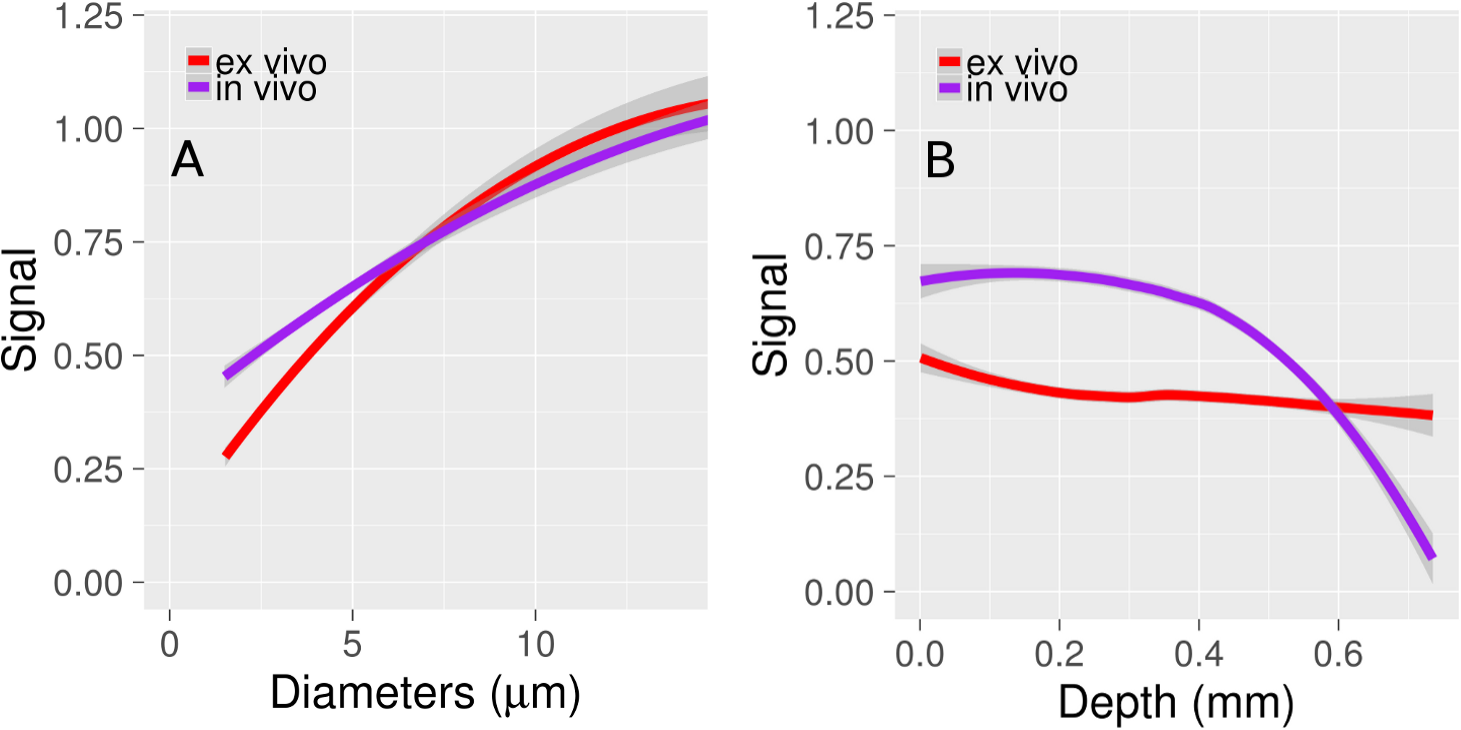
Vessel signal as a function of diameter and cortical depth. (A) Vessel signal as a function of diameter. Signal is normalized for the e*x* and *in vivo* data by calculating the mean signal of all vessels above 10 μm diameter in each of the 4 images. The signal for each vessel is calculated separately for each image. The mean signal for vessels above 10 μm diameter is given an arbitrary value of 1, and the signal for all vessels is calculated relative to this normalized value. Smaller vessels have a weaker signal *ex vivo* compared to *in vivo*, likely due to the larger PSF *ex vivo*. (B) Capillary signal as a function of cortical depth. The *in vivo* signal is constant for the first several hundred microns, before decreasing quickly with depth (characteristic attenuation length of 171 ± 15 μm). In contrast, the *ex vivo* signal maintains its strength through the cortical thickness. The lines in Figs A and B are fits to the data, and the ribbons surrounding the lines are the 95% confidence intervals.

The vessel signal as a function of diameter and depth is plotted in Fig 4A and 4B respectively. Signal increases as a function of vessel diameter (Fig 4A). The normalized signal is greater *in vivo* compared to *ex vivo* for vessels with diameters below 6 μm. Microvascular signal decreases with depth at a faster rate *in vivo* compared to the *ex vivo* data, with a relatively rapid drop-off beginning at about 400 μm below the cortical surface (Fig 4B). The characteristic attenuation length *in vivo* was 171 ± 15 μm across the four mice. This was calculated following the method of Kobat et al. [27], who measured the attenuation length to be 131 μm at 775 nm excitation, and 285 μm at 1280 nm. Axial slices at different depths are shown in S1 Fig for one of the *in vivo/ex vivo* data sets.

**Fig 5:**
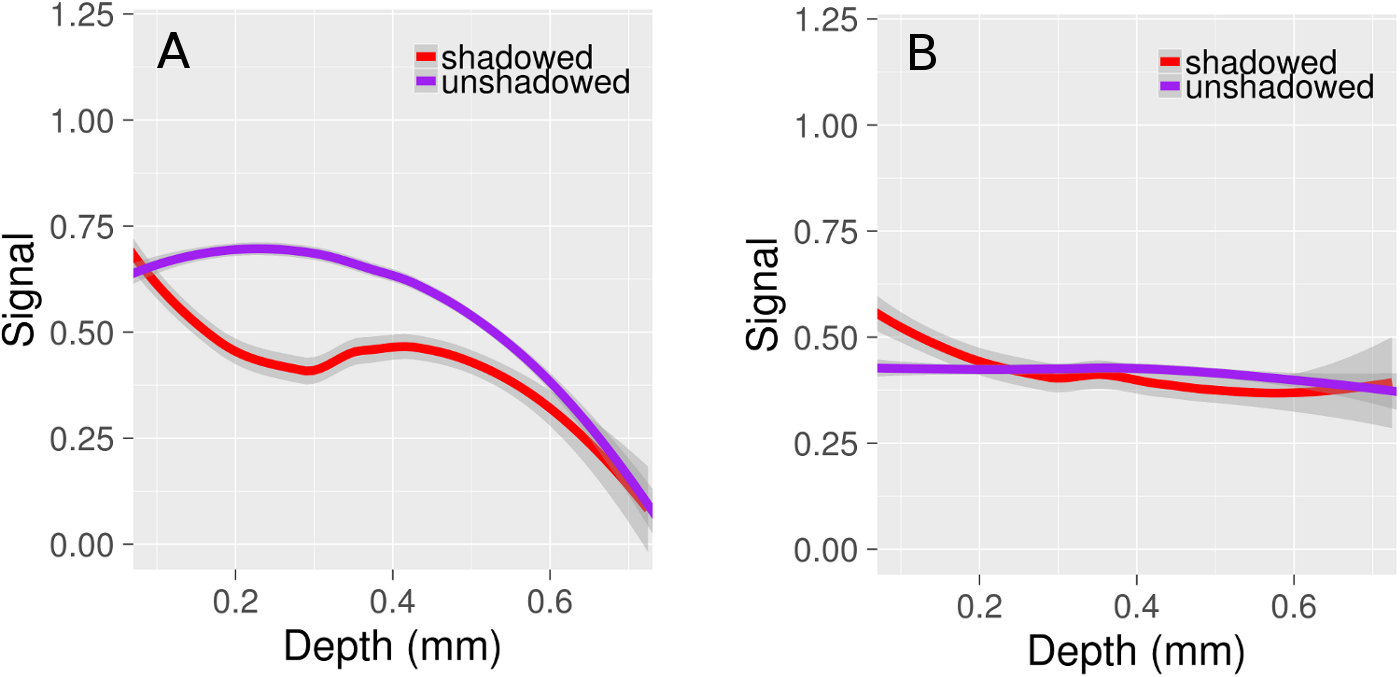
The impact of vessel shadowing on capillary signal. (A) *In vivo* (B) *Ex vivo*. The shadowing artifact is noticeably absent *ex vivo* (no difference in signal between shadowed/unshadowed vessels), but significant *in vivo* for depths below 0.6 mm.

Hemoglobin absorbs and scatters light, and its presence in large numbers in large-diameter pial surface vessels cause regions of an image beneath them to appear dark. This is termed a shadowing artifact, and often renders small structures beneath the cortical surface invisible under fluorescent microscopy [28]. Regarding visualizing the microvasculature, shadowing either blocks signals from capillaries or diminishes them entirely, and contributes to reduced imaging depth. Fig 5A plots the signal from shadowed and unshadowed microvessels, where shadowed vessels are defined as those located directly beneath a surface pial vessel. Although the signal from shadowed and unshadowed vessels *in vivo* is equally weak at depths greater than 0.6 mm, at shallower depths the difference is significant. The shadowing artifact is absent *ex vivo* (Fig 5B). The plots in Figs 4 and 5 were obtained by amalgamating the data from all vessels in the four pairs of images. Fitting was performed in R using Loess regression [29].

98.6 ± 0.7% (mean ± sem) of capillaries were successfully perfused by the fluorescent gel (average calculated over all four specimens). Unperfused capillaries possessed smaller diameters (3.74 ± 0.09 μm) compared to those that were perfused (4.12 ± 0.01 μm) (p = 0.0001; Student's t-Test; data pooled from all mice).

**Fig 6:**
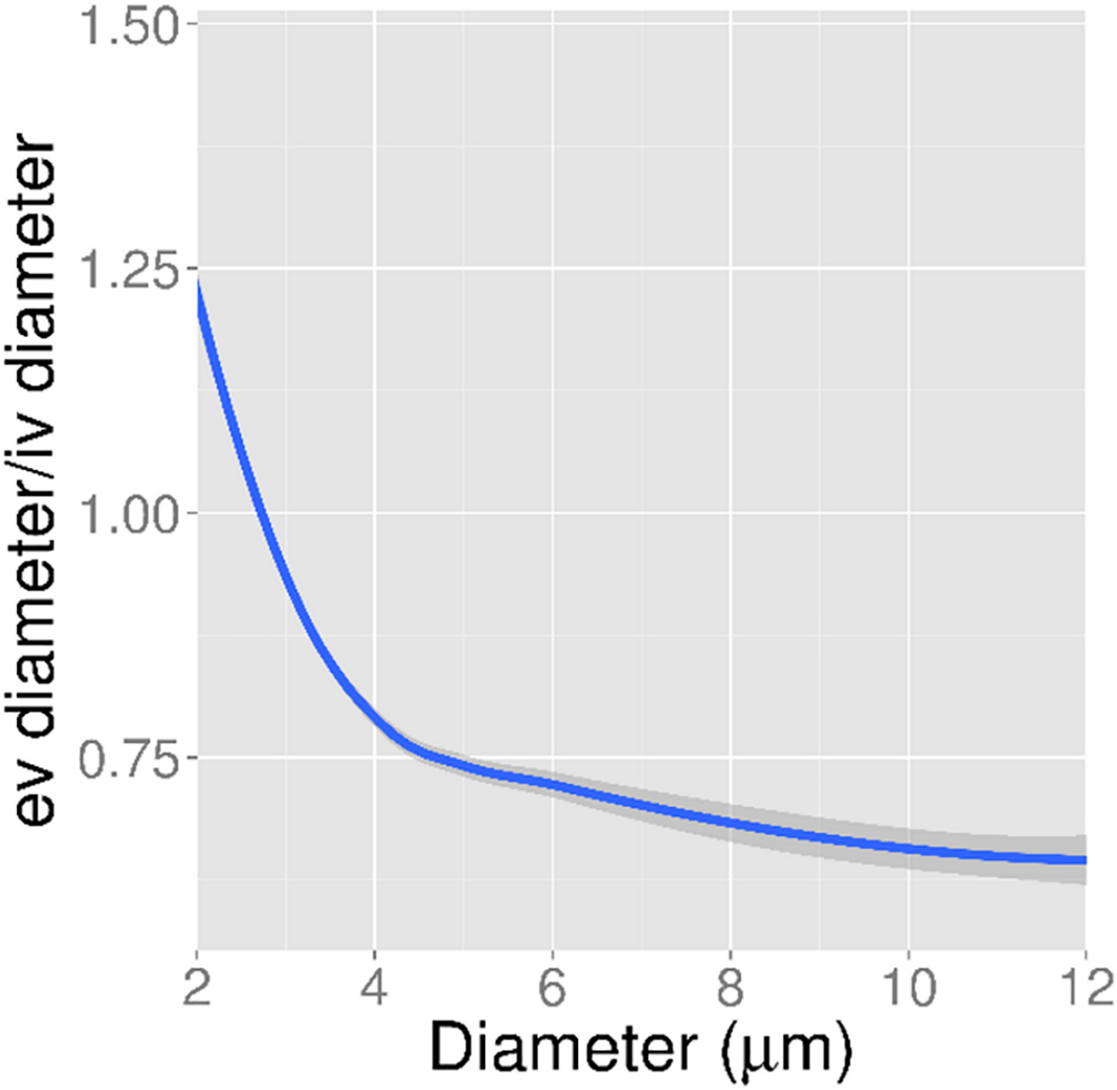
Ratio of *ex vivo*: *in vivo* vessel diameters as a function of *in vivo* vessel diameter. For each vessel, the ratio of its diameter *ex vivo* (after correction for refractive index mismatch) to that *in vivo* was computed. In this figure are the ratios computed for all vessels pooled together from the four mice.

Fig 6 demonstrates the extent of diameter shrinkage as a function of *in vivo* vessel diameter. *Ex vivo* capillary diameters throughout the cortex were slightly smaller than those measured *in vivo* (3.6 ± 0.5 μm vs. 4.2 ± 0.4 μm), but this difference was not statistically significant (p = 0.06; Student's t-Test) and varied between specimens (0% to 26%). In a one-to-one comparison of *in vivo* to *ex vivo* capillaries, 76% of capillaries were larger *in vivo* compared to *ex vivo* (p = 0.047). At the level of the penetrating vessels, the mean shrinkage was 34% over all mice. *In vivo*, the mean artery diameter (6 arteries) was 11.4 ± 0.7 μm, while the mean venule diameter (19 venules) was 8.9 ± 0.8 μm (p = 0.02). The ratio of *ex vivo* to *in vivo* penetrating vessel diameters did not differ significantly between arteries and veins across the 4 mice (0.69 ± 0.08 arteries; 0.63 ± 0.06 veins; p = 0.6). S2 Fig shows the vessel shrinkage *ex vivo* as a function of vessel diameter for each of the individual capillaries, arteries, and veins. Even though mean shrinkage is 31% for arteries and 37% for veins, this shrinkage is variable. Some veins, for example, undergo almost no shrinkage, while others undergo more than 50%. These diameter differences cannot be attributed to penetration of the glycocalyx by SR101, since vessel diameters *in vivo* measured with SR101 were indistinguishable at the resolution of the imaging system from those measured with FITC dextran (see S3 Fig).

## Discussion

This paper presents a methodology for *ex vivo* imaging and calculation of vessel diameter in the presence of spherical aberrations. It presents a unique method for comparing *in vivo* to *ex vivo* images through precise analysis of corresponding vessels in the two types of data. Novel *ex vivo* techniques are being continuously developed, and the methodologies demonstrated here can be applied to any variety of clearing materials or imaging modalities. Tsai et al. [17] previously compared vessel diameters between *in vivo* and *ex vivo* 2PFM. In their study, average diameters were compared in different mice, whereas in the present study, corresponding vessels from the same mice were compared. Their comparison with *in vivo* vessels was limited to vessels less than 100 μm below the cortical surface. In our study, the impact of pial vessels on capillary signal deep in the cortex was examined, together with the change in the microvascular signal as a function of depth and diameter. To accurately compare inner vessel diameters *in vivo/ex vivo*, the contrast agents used (either SR101 or the gel) must fill the lumen entirely without penetrating the glycocalyx or endothelial cell layers. FITC albumin is sufficiently large such that it does not penetrate the glycocalyx and only fills the lumen. Previous studies have demonstrated that SR101, a smaller molecule, is contained within the lumen and provides a reliable measurement of inner vascular diameter. For example, Sekiguchi et al. [30] estimated a difference of approximately 1 μm for pial artery diameters measured based on GFP-fluorescent endothelial cells versus those with plasma labelled with SR101. Further, Choi et al. [31] overlaid in vivo 2-photon vascular images of plasma-labelled SR101 and 2 MDa FITC dextran, demonstrating similarities in vessel diameters. Our own experiments indicate that there are no detectable diameter differences, at the resolution of our imaging system, between vessels labelled with either SR101 or FITC (see S3 Fig).

Past studies of brain microcirculation have used corrosion casting and/or histology. Due to the high resolution of electron microscopy and the 2D rendering of 3D surfaces, corrosion casting allows for a qualitative analysis of microvascular structure. Quantitative metrics, however, such as vessel length, rely on the angle at which the cast is viewed and are inconsistent between specimens if not accounted for [32]. In addition, because the tissue support is dissolved, finer vessels may break off the cast during corrosion, while imaging depth is limited unless the cast is dissected. In contrast, 2PFM with optical clearing enables visualization deep into tissue, without the need for dissection. As demonstrated here, the gelatin perfusate consistently fills more than 98% of the vasculature, whereas perfusion materials for corrosion casting are often more viscous. Since the tissue is not dissected, the brain is preserved for histological analysis at later time points. This may enable cellular-level tissue information to be correlated with vascular changes. Although the perfusion materials described here (2% gelatin with FITC-albumin) have been previously utilized, the authors Tsai et al. [17] combined this perfusion with clearing in 60% sucrose, resulting in an average imaging depth of 700 μm. Fructose, in contrast, may yield imaging depths up to 8 mm [15]. *Ex vivo* imaging depth in this study was only limited by the technical specifications of the microscope (2mm, the objective working distance) although we chose to image no deeper than the cortical depth, often a little over 1 mm. The mean *in vivo* imaging depth was 650 ± 45 μm, with a significant drop in contrast and signal at depths below 0.4 mm.

*Ex vivo* images may be significantly distorted when the refractive index of the clearing agent differs significantly from that of the immersion medium, typically water for long working distance microscope objectives. Multiphoton processes are strongly influenced by aberrations since absorption probability depends non-linearly on the focal intensity. These aberrations can be entirely eliminated through specialized lenses which use the clearing agent as an immersion medium [11, 15]. This route is costly, however, unlike the methodology presented in this paper.

Our methodology for accurately calculating vessel diameter has potential for application to other 3D microscope-based imaging techniques. Light sheet microscopy utilizes a thin sheet of light focused with a cylindrical lens to excite fluorescence within a sample, which is collected by a detection objective lens [33]. The PSF in light-sheet methods is obtained by combining the detection and illumination PSFs. This produces a system PSF with different FWHMs in the lateral and axial dimensions, similar to 2-photon [34]. Light sheet microscopy may be combined with optical clearing methods to image large samples, such as entire rat brains at micron-level resolution [35]. Due to the combination of excitation light-sheet generation with widefield detection, imaging of an entire mouse brain may be accomplished in under one week at μm-scale resolution [36]. Imaging of a similar volume of tissue with 2-photon would require months.

Capillary diameters were smaller on average by 13% *ex vivo* compared to *in vivo* (p = 0.06), although this shrinkage was non-significant, while 76% of capillaries were smaller *ex vivo*. One possible explanation is differences in pressure during perfusion at constant flow rates *ex vivo* in comparison with those in a live animal. An advantage of constant flow rate (as opposed to constant pressure) is that a flow rate sufficiently high may be selected to maximize the percent of vessels perfused. Craniotomies and tissue fixation may induce changes in tissue dimensions, such as swelling, leading to vascular compression. Despite these possibilities, mean capillary diameter differences between the two techniques is only 0.6 μm, while other techniques yield capillaries with comparable diameters to our reported values. As an example, India Ink perfusion techniques (India Ink dissolved in gelatin) yielded mean capillary diameters of 3.48 μm, with diameters ranging from 1.708 μm to 9.626 μm [37], while Tsai et al. [17] have reported an average diameter of mouse microvessels of 3.5–4.0 μm. These compare favourably with our mean capillary diameter of 3.6 μm. Although Tsai et al. [17] found vessel diameters *ex vivo* to match those *in vivo*, the *in vivo* measurements were performed on awake mice, whose diameters were not dilated by isoflurane. Their perfused mice were anaesthetized with pentobarbital (Nembutal), not ketamine/xylazine as in this study. The vasodilatory effects of barbiturates such as pentobarbital are well-known [38].

Shrinkage dependence on diameter was also demonstrated. This was possible due to the correspondence between *in vivo*-*ex vivo* vessels. To overcome the effects of shrinkage on vessel network quantification, diameters are often multiplied by a scaling factor [24]. Our findings suggest this may not be the optimal approach. Large diameter vessels may experience the most shrinkage, while the smallest capillaries may be larger *ex vivo* compared to *in vivo*. This finding may serve to caution researchers that *ex vivo* images should be calibrated at the outset to permit accurate diameter estimation.

Some of this shrinkage is attributable to the isoflurane anaesthesia *in vivo*. Isoflurane is a potent dilator of microvessels and induces breakdown of the blood-brain-barrier [39], whereas ketamine (the anaesthetic under which the mouse was perfused) has been shown to constrict arterioles in rat skeletal muscle by as much as 30% [40]. Under 1% isoflurane, capillary diameters relative to those exposed to ketamine/xylazine in cortex dilate by a factor 1.24, while under 3% isoflurane they dilate by a factor 1.85 [39]. Gao et al. [41] showed that 2% isoflurane dilates surface and intracortical arterioles by approximately 40%. The magnitude of these differences is comparable to our findings.

Factors beyond anaesthesia may influence in vivo/ex vivo diameter measurements. Arterial PCO2 affects vascular tone and can rise if an animal hypoventilates under anaesthesia. In addition, Navari et al. [42] have demonstrated that atmospheric exposure of the brain during craniotomy may reduce tissue CO2, resulting in arteriole constriction. Edema, which can be caused by craniotomy, may result in tissue swelling and increased intracranial pressure [43], leading to a compression of vessel diameters. Given these factors which bias estimates of vessel diameter under in vivo and ex vivo conditions, it is important that studies of disease or abnormal physiological states makes use of appropriate control conditions so that the differential effect of the intervention on vessel dimensions can be assessed. In general, estimation of vascular resistance from microvascular dimensions should be approached cautiously because small errors in vessel diameter are amplified when the corresponding vascular resistance is computed [5].

Despite a mean shrinkage of capillaries by 13%, this number is still sufficiently small such that the technique could be used to assess changes in vessel diameters in various disease states. For example, following 1 hour of reperfusion after global cerebral ischemia in gerbils (15-minute bilateral carotid artery occlusion), capillary and precapillary arteriole diameters are reduced by 30% and 24% respectively [44]. 24 hours following a fluid-percussion Traumatic Brain Injury, vessel diameters are reduced by 13% in injured cortex compared to non-injured cortex [45]. Although this is at the detectability limit of our in vivo-ex vivo comparison (mean shrinkage of capillaries of 13% according to our data), our use of isoflurane *in vivo* likely exacerbated the difference between ex vivo and in vivo diameters; use of a different anaesthetic *in vivo* would likely enable subtler detection of diameter changes.

Larger vessels exhibited a stronger signal both *in vivo* and *ex vivo* (Fig 4A). This is probably due to the volume of excitation being entirely encompassed within the vessel, resulting in an increased number of fluorescent particles detected. The relative signal is greater for capillaries *in vivo* compared to *ex* vivo, which we attribute to the increased PSF size *ex vivo*. Depending on the imaging depth, this size could be on the order of a capillary. Signal steadily decreases with cortical depth *in vivo* (Fig 4B), with a sharp drop-off at depths greater than 0.4 mm. In contrast, the signal remains relatively constant *ex vivo*. A constant signal with cortical depth is an advantage of *ex vivo* imaging.

*In vivo* data displayed a shadowing artifact which was absent *ex vivo* (Fig 5). This artifact is likely due to the higher amounts of hemoglobin in the large vessels. At a hematocrit of 45% and wavelength of 633 nm, the scattering coefficient of blood is about 80 /mm, while its absorption coefficient is about 0.8 /mm [46]. When the hemoglobin has been flushed from the vasculature during perfusion and is replaced with a transparent gel, the shadowing artifact disappears *ex vivo*. *In vivo* imaging is limited in the field of view chosen as regions covered by large pial vessels will be challenging to image; however, *ex vivo* has no constraints in imaging location. Haiss et al. [28] solved this dilemma *in vivo* by replacing the blood with a perfluorocarbon emulsion. This method, however, would constrain the contrast agents used for visualizing the vasculature as they must now be miscible with the emulsion. We found with our specimens little difference in the signal between shadowed and unshadowed vessels beyond 500 μm depth. It is still difficult to confirm from our work that pial vessels do not limit imaging depth, as none of the fields of view chosen contained a large cluster of pial vessels. Imaging depth would likely be different in such a region compared to one devoid of of pial vessels. For example, Haiss et al. [28] found that replacing blood with perfluorocarbon dramatically increases imaging depth below pial vessels and increases fluorescence intensity by almost a factor of 9. This suggests that in a region with a high pial vessel density, imaging depth is decreased by hemoglobin. While signal intensity varied across vessels in our study, all vessels present ex vivo were visible in vivo, regardless of location.

For all mice imaged, the perfusion success exceeded 98%, in agreement with Tsai et al. [17] who quoted a high success rate with the same perfusion protocol (close to 100%). The larger diameter of the perfused vessels compared to those unperfused suggest that perfused vessels had a lower resistance to fluid flow, and were more easily filled by the gel. This is in agreement with the Hagen-Poiseulle equation, where the resistance scales with the inverse of diameter to the 4^th^ power [5]. Thus, even a slight shrinkage in diameter may significantly impact the probability of a vessel being perfused.

## Conclusions

Differences between vascular morphology and signal in *in vivo* and *ex vivo* 2PFM are quantified in this study. Through development of a protocol that enabled analysis of the corresponding vessels in both image types, previously unexamined features of 2PFM, such as shadowing, were precisely quantified. Most studies do not image corresponding regions with separate imaging techniques, which limits the scope of their analysis. This study employed specific perfusion and clearing techniques; however, much of the data and analysis methods is applicable to a range of clearing materials and situations. The vessel tracking algorithm which accounts for spherical aberrations is applicable to any data set and combination of microscope objective and clearing materials, merely requiring acquisition of PSF data. The ability to accurately segment and quantify vessel properties is critical since for small capillaries the signal is weaker *ex vivo* compared to *in vivo*.

In conclusion, we have presented a novel imaging and analysis methodology for visualizing and analyzing the vasculature. This methodology outlined features of *in vivo* and *ex vivo* 2PFM, such as the influence of PSF on vascular signal, the impact of shadowing on microvascular signal, and the changes in vessel diameter. Overall, *ex vivo* imaging was found to be valuable for studying deep cortical vasculature.

## Supporting information

S1 Fig Slices at different depths through a specimen imaged with 2-photon. Left column is *in vivo*, right column *ex vivo*. Centerlines of vessels are shown in red. A and B are 450 μm below the cortical surface, while C and D are 650 μm below the cortical surface. Although the signal between *in vivo- ex vivo* is comparable at 450 μm, the contrast to noise ratio *ex vivo* is noticeably greater at 650 μm.
(TIF)

S2 Fig Shrinkage of arteries, capillaries, and veins as a function of diameter. Each scatter point (which represents a vessel segment) is labelled as an artery (red), capillary (green), or vein (blue).
(TIF)

S3 Fig Comparison of in vivo vessel diameters measured with SR101 and FITC dextran perfusion. (A) Image acquired on the FITC channel (B) Image acquired on the red (SR101) channel (C) Images A and B merged. The merging of the red and green channels produces a brown shading indicative of overlap between red and green signal. Because all vessels are entirely painted brown in C, it is concluded that vessel diameters *in vivo* measured with SR101 are indistinguishable at this resolution from those measured with FITC dextran. This suggests that any possible leakage of SR101 into the glycocalyx does not impact measurement of vessel diameter at the resolution of the imaging system.
(TIF)
