# Supplementary figures and images for: 3D morphological analysis of the mouse cerebral vasculature: Comparison of in vivo and ex vivo methods
Source: PLoS One. 2017 Oct 20;12(10):e0186676. doi: 10.1371/journal.pone.0186676 (PMC5650181; doi:10.1371/journal.pone.0186676)

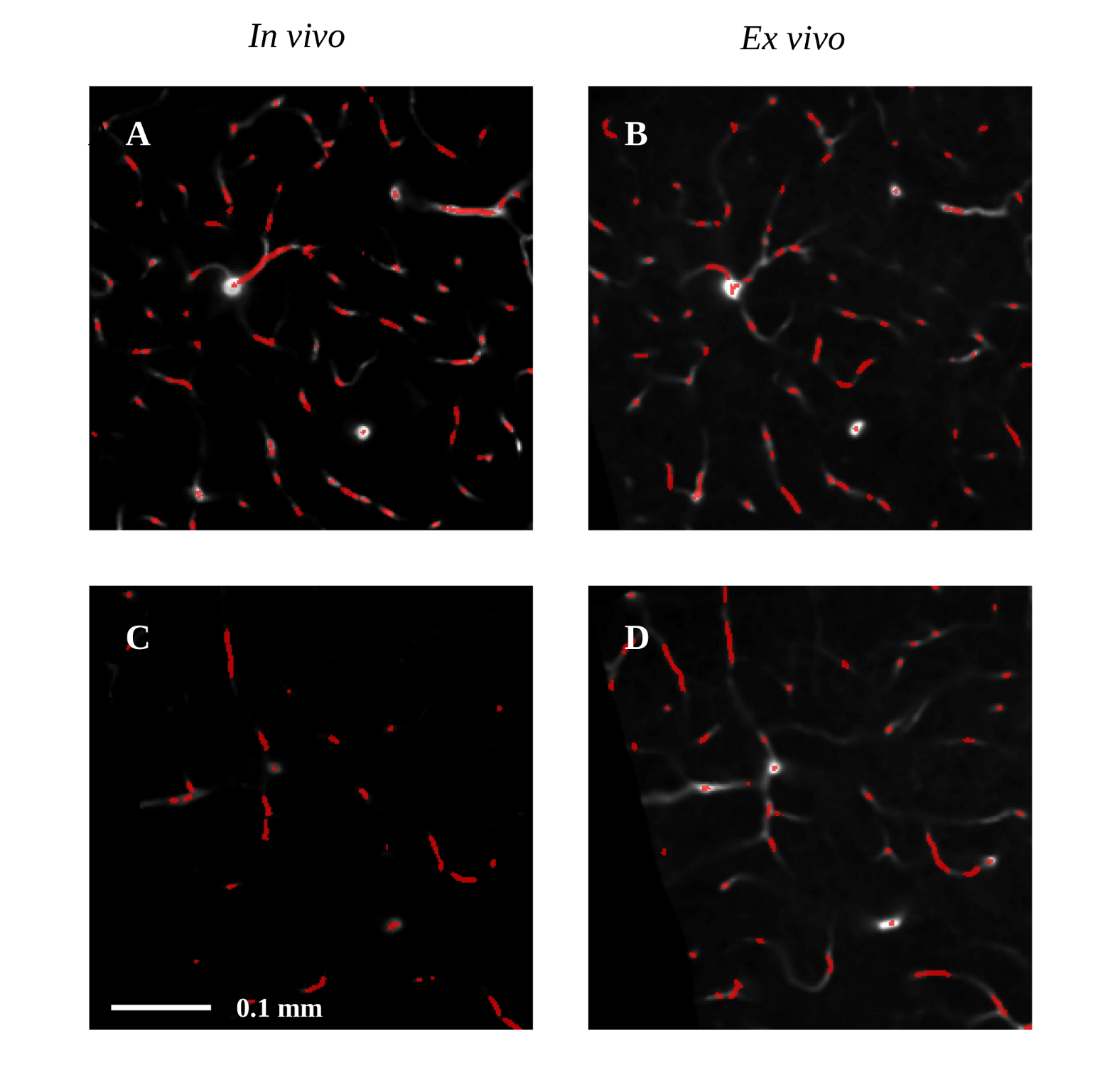

Supplement: S1 Fig — Left column is in vivo, right column ex vivo. Centerlines of vessels are shown in red. A and B are 450 μm below the cortical surface, while C and D are 650 μm below the cortical surface. Although the signal between in vivo- ex vivo is comparable at 450 μm, the contrast to noise ratio ex vivo is noticeably greater at 650 μm. (TIF) [file pone.0186676.s001.tif]

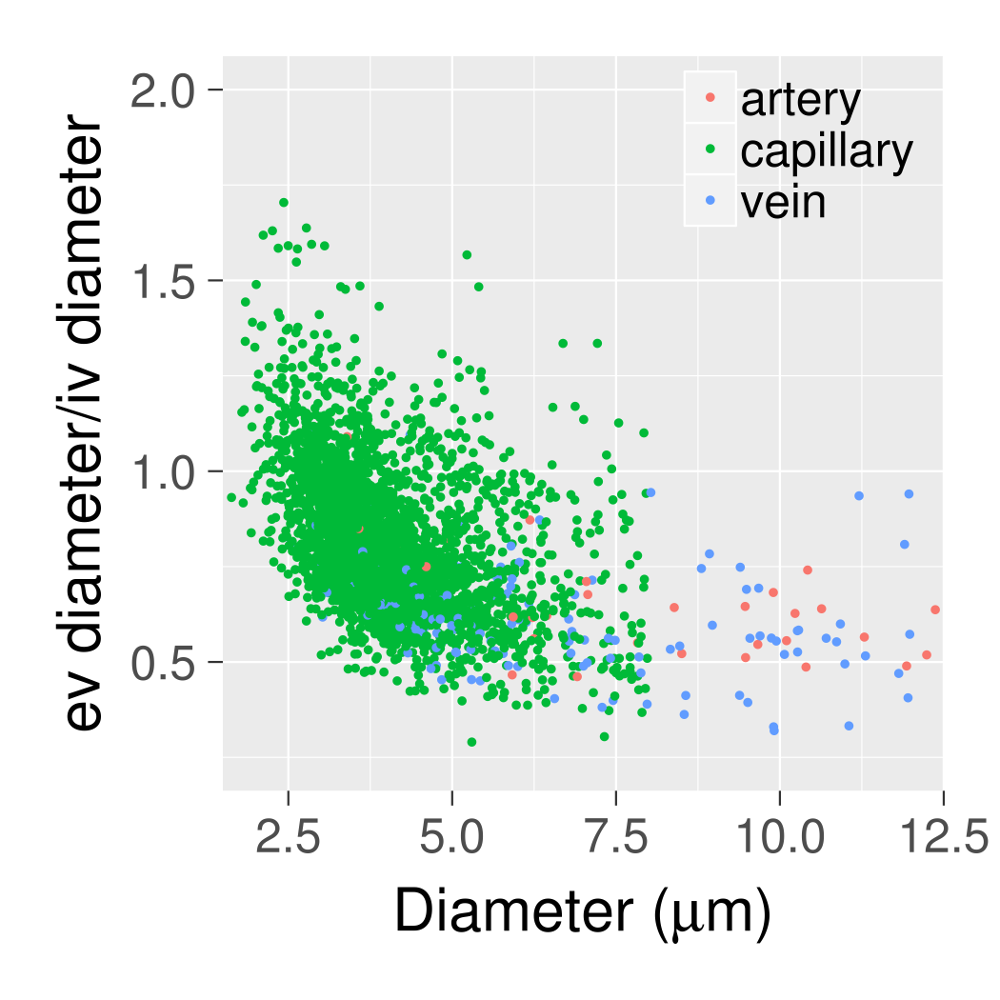

Supplement: S2 Fig — Each scatter point (which represents a vessel segment) is labelled as an artery (red), capillary (green), or vein (blue). (TIF) [file pone.0186676.s002.tif]

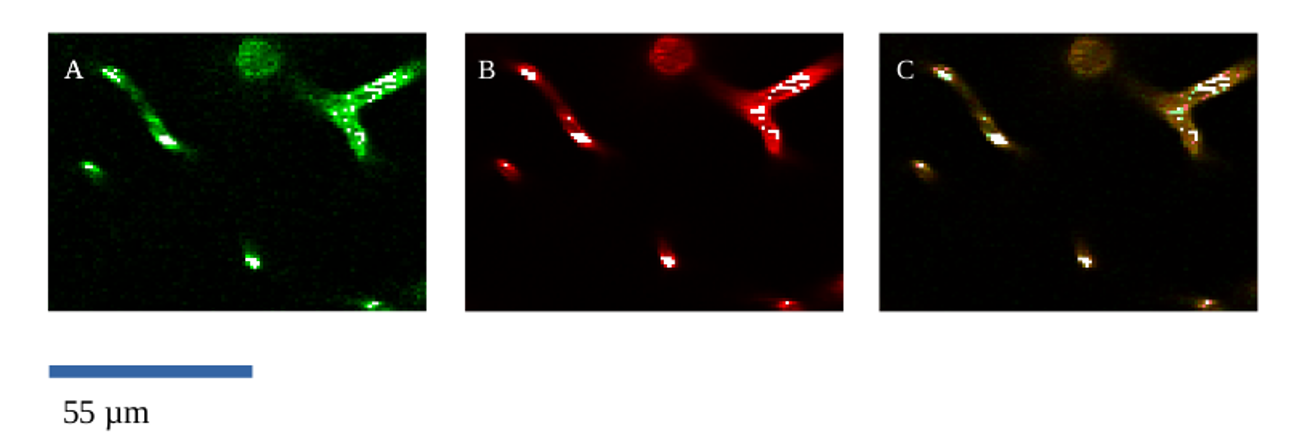

Supplement: S3 Fig — (A) Image acquired on the FITC channel (B) Image acquired on the red (SR101) channel (C) Images A and B merged. The merging of the red and green channels produces a brown shading indicative of overlap between red and green signal. Because all vessels are entirely painted brown in C, it is concluded that vessel diameters in vivo measured with SR101 are indistinguishable at this resolution from those measured with FITC dextran. This suggests that any possible leakage of SR101 into the glycocalyx does not impact measurement of vessel diameter at the resolution of the imaging system. (TIF) [file pone.0186676.s003.tif]
